# Child Feeding and Parenting Style Outcomes and Composite Score Measurement in the ‘Feeding Healthy Food to Kids Randomised Controlled Trial’

**DOI:** 10.3390/children3040028

**Published:** 2016-11-10

**Authors:** Kerith Duncanson, Tracy L. Burrows, Clare E. Collins

**Affiliations:** 1 Nutrition and Dietetics, School of Health Sciences, Faculty of Health and Medicine, the University of Newcastle, University Drive, Callaghan, NSW 2308, Australia; tracy.burrows@newcastle.edu.au (T.L.B.); clare.collins@newcastle.edu.au (C.E.C.); 2 Priority Research Center in Physical Activity and Nutrition, the University of Newcastle, Callaghan, NSW 2308, Australia; 3 117 Becker Road, Forster, NSW 2428, Australia

**Keywords:** child feeding practices, parenting style, domain, randomised controlled trial

## Abstract

Child feeding practices and parenting style each have an impact on child dietary intake, but it is unclear whether they influence each other or are amenable to change. The aims of this study were to measure child feeding and parenting styles in the Feeding Healthy Food to Kids (FHFK) Randomized Controlled Trial (RCT) and test a composite child feeding score and a composite parenting style score. Child feeding and parenting style data from 146 parent-child dyads (76 boys, aged 2.0–5.9 years) in the FHFK study were collected over a 12-month intervention. Parenting style was measured using parenting questions from the Longitudinal Study of Australian Children and the Child Feeding Questionnaire (CFQ) was used to measure child feeding practices. Data for both measures were collected at baseline, 3 and 12 months and then modelled to develop a composite child feeding score and a parenting score. Multivariate mixed effects linear regression was used to measure associations between variables over time. All child feeding domains from the CFQ were consistent between baseline and 12 months (*p* < 0.001), except for monitoring (0.12, *p* = 0.44). All parenting style domain scores were consistent over 12 months (*p* < 0.001), except for overprotection (0.22, *p* = 0.16). A significant correlation (*r* = 0.42, *p* < 0.0001) existed between child feeding score and parenting style score within the FHFK RCT. In conclusion, composite scores have potential applications in the analysis of relationships between child feeding and dietary or anthropometric data in intervention studies aimed at improving child feeding or parenting style. These applications have the potential to make a substantial contribution to the understanding of child feeding practices and parenting style, in relation to each other and to dietary intake and health outcomes amongst pre-school aged children.

## 1. Introduction

Child feeding practices and parenting style both impact on child dietary intake, but whether the influences are independent or related is of conjecture. Despite an increased focus of research on the influence of parents on child dietary intake, consistent relationships between child feeding practices and parenting style has not been established [1]. Uncertainty exists about whether child feeding practices and parenting style are amenable to change and the extent to which child feeding practices and parenting style are influenced by the changing needs of the child [2]. Adding to the complexity of measuring the child feeding practices and parenting styles is the personality of the children, the nature of parent–child interactions and the various strategies parents may use with their children to influence eating behaviour [3]. Child feeding practices are usually determined using a self-administered questionnaire, with the validated Child Feeding Questionnaire (CFQ) used most often [1,4]. Test-retest reliability of the CFQ has been established over a two-week period [4] but the reliability of the Child Feeding Questionnaire over time has not been routinely measured in intervention studies [2].

Moderate, but consistent evidence exists for a negative association between restriction of food and rebound overeating, with subsequent high child Body Mass Index (BMI) [5,6,7]. Pressuring a child to eat has been reported to have an inverse impact on dietary intake, and has been linked to reduced food consumption and fussy eating behaviours [7,8,9,10,11]. In another study, such pressuring behaviours predicted higher fruit and vegetable intake by children [12], suggesting that the same behaviours may result in either positive or negative child dietary intake outcomes depending on the food type.

Parental responsibility for child dietary intake [13,14] and monitoring of consumption, particularly in relation to discretionary foods [6,13,14,15] have been consistently associated with healthy eating behaviours and weight status in children. However, the measurement of some child feeding practices can be challenging due to the complexity of concepts. For example, parental control over eating might be considered overt or covert, and these practices may differ in their relationship with child dietary intake or impact on weight status [16]. In general, the existing evidence suggests that improved child dietary intake and lower risk of child overweight are associated with high parental responsibility for feeding and monitoring of food [17], and with low pressure to eat and low overt food restriction [18].

The child feeding practices associated with the provision of food to children [4] have been assumed to change in the early years as both child development [19,20] and parenting practices evolve [5]. One of the few studies to assess continuity of child feeding practices from child age two to five years using the CFQ showed increased maternal pressure to eat over three years with mean score of 2.10 (0.67) to 2.79 (0.75), *p* < 0.01 [2]. However, stability in measures of parental restriction (mean 3.27 to 3.25) and monitoring (mean 4.27 to 4.46) of food intake were displayed [2]. An improved understanding of relative stability or change in child feeding practices of parents of preschool aged children and whether any changes are related to the changing needs of the children would be a valuable contribution to this field of research.

Significant decreases in CFQ domain scores (*p* < 0.01) for restriction and pressure to eat, and increased monitoring provides evidence that specific child-feeding domains are modifiable and that changes can be sustained over time by parents of children aged over five years, in the context of a targeted obesity intervention [21]. Equivalent results amongst study samples of children aged under five years have not been reported.

Parenting styles have been conceptualised in terms of quantity and quality within two underlying dimensions of demandingness and responsiveness [22,23]. Demandingness refers to the extent to which parents show behavioural control and supervision in their parenting [23]. Responsiveness refers to the extent to which parents show affective warmth, acceptance and involvement [23]. Parenting style has generally been reported as being stable over time [24], although components such as parenting sense of competence [25] and self-efficacy [26] may be amenable to change through general parenting interventions. Direct associations between parenting style and child eating behaviours have not been investigated to the same extent as those mediated by the child feeding practices of parents. Generally, less warm, more hostile parent relationships are associated with children having less adaptive eating behaviours [27]. Maternal parenting warmth has been identified as a significant negative correlate of child BMI [9]. A combination of emotional warmth and high expectations for child dietary adequacy has been shown to positively impact on child fruit and vegetable consumption [28]. There is limited and conflicting evidence regarding the association between parenting styles and the child feeding practices of parents [29,30,31]. A recent systematic review [1] identified only seven studies that measured associations between child feeding and parenting style, despite fourteen other studies analysing both measures individually. Six of the seven studies identified at least one association between parenting style and a specific child feeding practice.

The available evidence indicated that pressuring a child to eat and adopting restrictive parental child feeding practices occurs in association with an authoritarian parenting style, characterised by a high level of demandingness, but low responsiveness [30,31]. Conversely, the use of monitoring by parents to keep track of child food intake is more likely to occur in association with higher scores for parental warmth [32]. Similarly, an authoritative parenting style has been positively associated with both monitoring of child dietary intake and parental responsibility for feeding [30]. These results suggest that supporting parents to adopt low demandingness and high responsiveness around feeding may provide the ideal environment for children to develop internal eating cues to eating [33,34].

Permissive parenting style is associated with less support, control and structure. Whilst the absence of controlling practices may result is lower food restriction [31], a lack of monitoring of child food intake may result in an unbalanced diet [30,31]. Recent investigation in this field [10] has focused on the influence of contextual parenting factors in moderating the relationships between food parenting and child dietary behaviour. For example, associations of encouragement and covert control with desirable child dietary behaviours were found to be stronger for children who were reared in a positive parenting context [10].

A major gap in the literature examining parenting style and child feeding practices is the relative absence of reporting of the associations between these measures, when compared to reporting of either measure alone in relation to child nutrition or weight status outcomes. Three large scales intervention studies [6,35,36] used the CFQ and consistent parenting style tools, but the association between the two measures was not reported.

Specific child feeding practices and the components of parenting styles contain domain specific content that has been validated at a construct level [4,37]. To date this information has been used to compare domains with child dietary intake and health outcomes [5,6,7,8,9]. There is potential to consider weighting each domain based on respective known influences on child health and combine the results to form a single numeric measure that could be used as an indicator of overall child feeding competence, with an equivalent method and measure for parenting style.

The Feeding Health Food to Kids (FHFK) Randomised Controlled Trial (RCT) aimed to determine whether provision of high quality child feeding, parenting and nutrition information to rural parents using self-directed technology-based education resources had an impact on the dietary patterns of rural Australian children aged two to five years, or the child feeding practices and parenting style of their parents [38]. Impact on dietary patterns have been reported previously, with changes in consumption frequency for core and non-core food intakes resulting in a reduction in total energy intake, but no change in the proportion of total energy intake from non-core foods [38].

Therefore, the aims of the current study are three-fold. The primary aim is to determine intervention effects on child feeding and/or parenting style from the FHFK RCT. The second aim is to measure the stability of child feeding practices and parenting style dimensions in the FHFK study over 12 months. The final aim was to determine whether a correlation exists between a consolidated child feeding score and consolidated parenting style score, calculated from weighted individual child feeding and parenting style domain scores in the FHFK RCT study.

## 2. Methods

The full methodological details of the Feeding Healthy Food to Kids (FHFK) randomised control trial (RCT) have been published previously [38,39] and the trial registered, ACTRN12609000356268. Approval for the study was obtained in March 2009 from Hunter New England Human Research Ethics Committee. Reference Number: 08/12/17/4.02.

Briefly, parents of young children were recruited from child care facilities in five rural, low socio-economic localities in New South Wales, Australia. Inclusion criteria were: eldest child in family aged two to five years, without a chronic health condition that impacted on dietary intake. A child was excluded if they had a chronic disease, such as coeliac disease or a food allergy, that impacts significantly on dietary intake or if they had an older sibling in the study. Demographic data was obtained by questionnaire at baseline, with categorical responses reported by age group, education level and Aboriginal Torres Strait Islander status of parents and children, type of child care and child health status.

### 2.1. Child Feeding and Parenting Style Intervention

The Feeding Healthy Food to Kids RCT aimed to improve child feeding practices of parents and the dietary intake of their two to five year old children through dissemination of the Tummy Rumbles interactive CD [40] and Raising Children DVD [41] to be used over a 9 month intervention period from September 2009. In order to simulate likely population level resource dissemination, the only prompting provided to parents to use the resources was a reminder note delivered by post with the 3-month follow up surveys.

Intervention group participants were considered to have adhered to the study protocol if they reported using both Tummy Rumbles and Raising Children for at least one hour each during the intervention period. The contents and rationale for using Tummy Rumbles and Raising Children have been described [38,39]. Briefly, the Tummy Rumbles interactive nutrition education CD is a self-directed resource that was adapted from an early childhood nutrition education program for childcare staff and parents. The resource is divided into modules that include: the five food groups, dietary fats, fussy eaters, healthy lunchbox ideas, food budgeting and reading food labels.

Raising children is ‘a guide to parenting from birth to 5’, the content of which is based on the principles of the Raising Children website, Australia’s definitive parenting resource [41]. Intervention group participants in this study were requested to view the DVD’s child section, specifically the segments on eating strategies, junk food, encouraging behaviour, minimising choking risk, play and learning.

### 2.2. Child Feeding Practices Measurement

Parents completed the self-report, 31-item CFQ [4] at baseline, 3 and 12 months to identify whether the provision of self-directed parenting and child feeding resources influenced child feeding practices of parents or the dietary intake of their children. The CFQ is designed for use by parents of children aged 2 to 12 years. Items are measured using a five-point Likert-type scale, with each point on the scale represented by a word anchor. Parental beliefs and attitudes regarding child feeding practices are measured in seven domains; perceived responsibility (mean of three items), parent perceived weight (mean of four items), perceived child weight (mean of three items), parents’ concerns about child weight (mean of three items), monitoring (mean of three items), restriction (mean of eight items), pressure to eat (mean of four items).

Scores for each question in each of seven domains were aggregated and divided by the number of questions in each respective domain. The minimum domain score is one and maximum is five.

### 2.3. Development of Child Feeding Score

Child feeding outcome measures used in this study were perceived responsibility (RESP), monitoring (MON), restriction (REST) and pressure to eat (PTE). Based on existing literature responsibility [13,14] and monitoring [6,13,14,15] were positively weighted, with pressure to eat [7,8,9,10,11] and restriction [18] negatively weighted by adding one to maximum scores, and then subtracting the domain score. For example, a PTE score of 4 would have become (6 − 4) = 2. In order to develop an overall child feeding score, the following calculation was used:

Child feeding score (CFS) = RESP + MON + (6 − PTE) + (6 − REST)

The minimum child feeding score would be 4 and maximum score would be 20.

### 2.4. Parenting Style Measurement

The Longitudinal Study of Australian Children (LSAC) [37,42] parenting questions were used to evaluate parenting across a variety of domains. All six domains from the parenting section of the LSAC dataset were analysed in the FHFK RCT. Warmth, inductive reasoning, parental efficacy and overprotection were assessed using a 1–5 point Likert scale with responses ranging from ‘never/almost never’ (=1) to ‘always/almost always’ (=5). Warmth is a six-question domain that identifies the level of affection that a parent displays towards their child. Inductive reasoning is a three-question domain that analyses a parent’s level of communication or reasoning about the consequences of behaviours to a child, whilst setting reasonable limits on behaviour. Parental efficacy is a four-question domain that identifies child behaviours related to parental discipline. Overprotection is a three-question domain that identifies how much a parent attempts to shelter their child.

Self-efficacy is measured by a single question that asks parents how they feel, using a five-point Likert scale ranging from ‘a very good parent’ (=1) to ‘not very good at being a parent’ (=5). Parental hostility is five-question domain that assesses how often parents display anger related behaviour towards their child. This domain is measured on a ten-point Likert scale ranging from ‘not at all’ (=1) to ‘all the time’ (=10).

Scores for each question in each of the four selected domains were aggregated and divided by the number of questions in each respective domain.

### 2.5. Development of Parenting Style Score

For the current study, parenting outcome measures were: warmth (W), inductive reasoning (IR), parent efficacy (PE), self-efficacy (SE), overprotection (OP) and hostility (H). Based on existing literature [37,42,43] inductive reasoning, parent efficacy, self-efficacy and warmth were positively weighted. Overprotection was negatively weighted by adding one to maximum scores, and then subtracting the domain score. Parental hostility scores were negatively weighted by adding two to maximum score, subtracting the domain score, and then dividing the resulting figure by two, because the scale was 1–10 instead of 1–5. For example, a hostility score of six out of ten would have resulted in a weighted score of (12 − 6)/2 = 3. In order to develop an overall parenting score, the following calculation was used:

Parenting Style Score (PSS) = W + PE + SE + IR + (12 − H)/2 + (6 − OP)

The minimum score would be 6 and the maximum score would be 25.5.

### 2.6. Statistical Analysis

Descriptive statistics were reported as mean (SD) or mean difference (5% confidence interval (CI)). The mean scores for participants across each domain of child feeding and parenting style were calculated at baseline, three and twelve months post intervention. The data was assessed for normality of domain using logistic regression tests. Internal consistency was assessed using Cronbach’s alpha for items within each of the seven domains of the CFQ and six components of the LSAC parenting questionnaire. Paired t-tests were conducted to detect changes to domains of parenting and child feeding between baseline and 12-month follow up for the intervention group, the control group and across both groups combined. Pearson’s correlations adjusted for type 1 error by applying Holm Bonferroni correction were performed to examine if there was a relationship between domains of parenting and child feeding practices at baseline, three and twelve months follow up across both groups. Results from intervention and control groups were combined for the remaining analysis. Multivariate correlation testing was performed to measure reliability of each domain of each tool over time and multivariate mixed effects linear regression was used to measure associations between variables over time. Pearson’s correlations adjusted for type 1 error were performed to examine if there was a relationship between parenting and child feeding practices at baseline, three and twelve months follow up across both groups. Statistical significance was set at *p* < 0.05. Statistical analysis was completed using STATA statistical software (Version 10, College Station, TX, USA).

## 3. Results

### 3.1. Demographics

A total of 180 parent–child dyads were assessed for eligibility, with 146 randomised to intervention (*n* = 75) or control groups (*n* = 71). The demographic data (Table 1) indicates that there were no differences at baseline in parent or child characteristics, other than the percentage of parents over thirty years being significantly higher in the control compared to intervention group (83% vs. 66%, *p* < 0.05). The retention rate was 79% (*n* = 116) at 3 months and 60% (*n* = 87) at 12 months. Although this study was conducted in low socio-economic rural areas, the study cohort was of relatively high educational attainment, with 56% commencing tertiary studies. The mean child age of four years at study commencement aligned with a mean child age of five years at study completion.

### 3.2. Child Feeding Domains and Parenting Style Dimensions at FHFK RCT Baseline

There were no significant differences between the control and intervention groups for any child feeding domains and parenting style dimensions at baseline (Table 2), with the exception of monitoring, which was significantly higher for the control group 4.6 (0.52) than the intervention group 4.2 (0.76), *p* < 0.05. As shown in Table 2, all internal consistencies were above 0.7 except over-protection (0.69).

### 3.3. Changes to Child Feeding Practices and Parenting Style over 12 Months

Table 3 shows that within-group changes in individual domains of child feeding and dimensions of parenting style were not significantly different from baseline at 3 and 12 months post intervention.

In the intervention group at 12 months, warmth had significantly increased by 0.19 (95% CI 0.04, 0.35), *p* = 0.02 from baseline and overprotection increased by 0.29 (0.06, 0.53), *p* = 0.02. No statistically significant changes were identified in the control group (Table 3). Random effects analysis found no significant intervention effects of the FHFK RCT for any individual child feeding domains (*p* values 0.31 to 0.98) or parenting style dimensions (*p* values 0.13 to 0.83) (Table 3). The data from intervention and control groups was therefore pooled for all further analysis.

Pooled control and intervention group FHFK RCT data was used to conduct multivariate mixed effects linear regression analyses for child feeding domains and parenting style dimensions across all three time-points. Table 4 demonstrates that statistically significant associations were identified between warmth and both responsibility, 0.132 (0.02, 0.24), *p* = 0.020 and monitoring 0.15 (0.05, 0.24), *p* = 0.006. Inductive reasoning with responsibility 0.14 (0.03, 0.25), *p* = 0.017 *, Pressure to eat, 0.13 (0.01, 0.24), *p* = 0.033 and monitoring, 0.15 (0.05, 0.26), *p* = 0.006.

### 3.4. Correlation of Individual Child Feeding Domains and Parenting Style Dimensions over 12 Months

The stability of child feeding domain and parenting style dimension measures were confirmed using correlation analysis of pooled control and intervention group data. All child feeding domains were correlated between baseline, 3 months and 12 months (*p* < 0.05) with the exception of monitoring which showed no significant correlation between baseline and 3 months (0.26, 0.06) or baseline and 12 months (0.12, *p* = 0.44) (Table 5). All parenting style dimensions were correlated between baseline, 3 months and twelve months (*p* < 0.05) with the exception of overprotection which was showed correlation between baseline and three months (0.60, *p* < 0.0001) but not between baseline and 12 months (0.23, *p* = 0.16) (Table 5).

### 3.5. Correlation between Child Feeding Score and Parenting Styles Scores

Correlation was maintained between the overall total “Child Feeding Score” at baseline and 3 and 12 months (*p* < 0.001). Correlation between “Parenting Style Score” at all timepoints decreased from *p* < 0.0001 to *p* = 0.05 between the three and twelve month time points (Table 6). A strong correlation between overall child feeding score and parenting style score in the Feeding Healthy Food to Kids study was shown for the whole FHFK sample when measures for all timepoints were included (*n* = 314) (Table 6).

## 4. Discussion

The aims of the current study were to describe and determine the interventions effects of the child feeding practices and parenting style characteristics within the FHFK RCT over 12 months. Secondly, we used the FHFK RCT data to test composite scores derived from child feeding practices and parenting style domain scores. It was found that the baseline child feeding and parenting style characteristics of the FHFK RCT participants compared favourably to the characteristics reported in previous cross-sectional studies [2,43]. Individual child feeding practice domain scores of parents of children four to five years participating in the FHFK RCT were relatively high for perceived responsibility (mean 4.4 out of 5) and monitoring (mean 4.4 out of 5), moderate for restriction (3.4 out of 5) and low for pressure to eat (2.4 out of 5) when compared to reported results from previous studies in slightly different population groups [2,29,44]. Parenting style dimension means for warmth, inductive reasoning and parental efficacy were high compared to a nationally representative sample of children [43], which may be related to the relatively high educational attainment of the FHFK RCT participants.

Of 24 potential associations between parenting style dimensions and child feeding practice domains, only five associations were found to be statistically significant. Warmth and inductive reasoning each correlated independently with both monitoring and responsibility. Warmth and inductive reasoning are considered characteristic components of an authoritative parenting style, and therefore associated between these dimensions and monitoring and responsibility are consistent with existing research [1]. However, the association between inductive reasoning and pressure to eat was unexpected, highlighting the lack of consistency in associations between individual child feeding practices and parenting style dimensions [1].

The consistency of child feeding results across time and between study groups in the FHFK RCT indicate that child feeding practices of parents are relatively consistent, and the CFQ tool is reliable over a 12-month time frame for parents of children four to five years. These results approximate the findings of Farrow and Blissett [2] in the only previous study investigating the stability and consistency of child feeding in the pre-school age bracket. If parental usual child feeding practices are stable during the pre-school years, then changes to child feeding practices in well-designed research studies would be attributable to the intervention.

The absence of an intervention effect on any individual child feeding domain or parenting style dimensions may be partly attributable to the desirable baseline profiles of these variables within the FHFK RCT. In turn, these desirable child feeding practices and parenting style features may be partly attributable to the relatively high educational attainment and mean age of participating parents [43,45]. A further explanation could be that changing child feeding and parenting behaviours in a community level intervention, even when parents are provided with appropriate, evidence-based resources that they report using and valuing [45] requires a more targeted intervention.

Theoretically, the parenting style and child feeding practices should show stronger and more consistent associations, and the lack of evidence for this in the literature [1] and in the FHFK RCT may be indicative of a lack of sensitivity of tools and analysis or a research gap.

The value in replacing individual domain scoring with scores that reflect the overall child feeding or parenting style profile of the parent is supported by analysis of the child feeding score and parenting style scores. The correlation values for the CFS at 3 and 12 months (0.56 and 0.47) and PSS (0.45 and 0.22) were high relative to those between individual domains and dimensions from the FHFK RCT [45]. In comparable studies, correlation values ranged from 0.15 to 0.33 [1]. Further analysis of the child feeding score relative to child dietary intake within the FHFK RCT is recommended, particularly in relation to consumption of energy-dense, nutrient-poor foods and vegetables.

The current study indicates that the use of a composite score that reflects overall child feeding practices, and an equivalent score summarising parenting style dimensions are warranted. While not intended to replace existing validated tools that are used to classify child feeding practices and parenting styles, a numeric score that is a single continuous variable could be prove to be a versatile measure in studies investigating associations between child feeding practices or parenting style of parents with each other or with other measures of child health. Further refinement of the composite scores by utilising data sets from large, cross-sectional studies that have used the CFQ and LSAC [42] from which the parenting style dimensions were drawn, in order to achieve optimal weighting of variables to achieve good fit, and further testing for validity, internal and test-re-test reliability could be conducted in the future. These existing studies have large enough sample sizes for retrospective analysis to be conducted to test and validate the composite scores and then evaluate scores against child health outcomes.

The second major implication for practice is that child feeding practices of parents with pre-school aged children may be more stable than previously assumed or predicted. Given that the majority of studies in this age group that address both child feeding and parenting styles to date are cross-sectional in nature, this is an area which would benefit from considerable investment of research.

The cross-sectional nature of studies in the pre-school aged population, and primary school target groups for the vast majority of intervention studies that measure child feeding practices of parents, make it difficult to compare findings for interventions aimed at changing child feeding practices of parents with pre-school aged children.

The benefit of drawing data from a randomised controlled trial with a moderate sample size of 146 was diminished by the 40% (*n* = 66) drop-out rate throughout the study period. Two factors that potentially contributed to this were the demographic of the study being busy mothers of young children and the high participant burden associated with required assessments [38].

The pooling of data from the control and intervention groups for analysis of paired data after it was found that there was no intervention effect was a limitation of the study. The benefit of additional data pairs and nature of the second stage analysis for comparison between child feeding and parenting style scores were considered reasonable justification for this analysis.

The calculations for child feeding score and parenting style score were only weighted as positive or negative based on existing literature in the field [1]. These tools need to be weighted more sensitively following statistical modelling and further factor analysis prior to use in future studies. The associations identified between parenting style dimensions and child feeding practice domains may have resulted from the process of conducting multiple analyses rather than true statistical significance.

The parenting style score is further limited in application because the dimensions used have been taken from a population level nation-wide survey, rather than from a validated parenting style measurement tool. It would be valuable to conduct a similar analysis using one of the more widely used parenting style tools such as the Parenting Styles and Dimensions Questionnaire [31,46].

## 5. Conclusions

Child feeding practices were stable over a 12-month period and the CFQ tool was a reliable measure of child feeding, suggesting that child feeding practices may be less amenable to change than previously predicted. The correlation within composite child feeding practices and parenting style scores over 12 months and between these two variables suggest that composite scores have potential applications in the analysis of relationships between child feeding and dietary or anthropometric data, but require further refinement and validation before widespread use. Once validated, composite scores could be applied retrospectively to existing large datasets, or prospectively in intervention studies aimed at improving child feeding or parenting style. These applications have the potential to make a substantial contribution to the understanding of child feeding practices and parenting style, in relation to each other and to dietary intake and health outcomes amongst pre-school aged children.

## Figures and Tables

**Table 1:** Demographic characteristics of Feeding Healthy Food to Kids study parent and child participants at baseline by group.

Child Characteristics	Intervention (*n* = 75)	Control (*n* = 71)	Parent Characteristics	Intervention (*n* = 75)	Control (*n* = 71)
Child health			Parent age		
• no chronic condition	74 (99%)	69 (97%)	• Under 30 years	20 (34%)	12 (17%) *
• health condition	1 (1%)	2 (3%)	• 30 years and over	56 (66%)	59 (83%) *
Child care			Parent education		
• in some form of care	69 (92%)	65 (91%)	• Secondary educated	33 (44%)	33 (47%)
• no formal care	6 (8%)	6 (9%)	• Tertiary educated	42 (56%)	38 (53%)
Child gender			Parent gender		
• male	40 (53%)	37 (52%)	• Male	0 (0%)	1 (1%)
• female	35 (47%)	34 (48%)	• Female	75 (100%)	70 (99%)
Child indigenous status			Parent indigenous status		
• Aboriginal	2 (3%)	3 (4%)	• Aboriginal	1 (1%)	2 (3%)
• Not Aboriginal or Torres Strait Islander	73 (97%)	68 (96%)	• Not Aboriginal or Torres Strait Islander	74 (99%)	69 (97%)
Mean child age years (SD)	4.00 (0.13)	4.04 (0.91)	All results are *n* (%) unless stated		

* *p* < 0.05; SD: standard deviation.

**Table 2:** Comparison between groups at baseline for child feeding practices and parenting style in the Feeding Healthy Food to Kids Randomised Controlled Trial (*n* = 146).

Child Feeding Domains						Parenting Style Dimensions				
Group	Responsibility	Restriction	Pressure to Eat	Monitoring	Warmth	Parental Efficacy	Self-Efficacy	Inductive Reasoning	Hostility	Overprotection
Total (*n* = 146)	4.4 (0.59)	3.4 (0.85)	2.5 (0.89)	4.4 (0.67)	4.6 (0.38)	3.1 (0.29)	1.9 (0.78)	4.3 (0.63)	3.2 (1.30)	3.7 (0.68)
Internal consistency	0.88	0.80	0.71	0.89	0.85	0.82	NA	0.75	0.85	0.62
Intervention (*n* = 75)	4.4 (0.66)	3.4 (0.93)	2.5 (0.89)	4.2 (0.76)	4.7 (0.33)	3.2 (0.25)	1.9 (0.76)	4.3 (0.54)	3.2 (1.25)	3.7 (0.67)
Control (*n* = 71)	4.4 (0.53)	3.6 (0.74)	2.7 (0.89)	4.6 (0.52) *	4.6 (0.40)	3.1 (0.33)	1.9 (0.81)	4.3 (0.71)	3.2 (1.35)	3.8 (0.69)

All measures range from 1 to 5, except hostility (1–10); All data is displayed as mean (SD); * *p* < 0.05 (group effect); Internal consistency measured using Cronbach’s alpha; NA: Not applicable (one variable only).

**Table 3:** Intervention effect of the Feeding Healthy Food to Kids Randomised Control Trial on child feeding and parenting style dimensions.

Outcome	Time Month	Group Allocation				Mean Difference between Groups		Group × Time
		Control (*n* = 71)	*p* Value	Intervention (*n* = 75)	*p* Value			
		Mean Change from Baseline ((95% Confidence Interval)) ^a^						
Child Feeding Practices						(95% CI)	*p*	*p* Value
Responsibility	3	0.05 (−0.18, 0.09)	0.48	0.02 (−0.11, 0.14)	0.81	0.07 (−0.15, 0.28)	0.54	
	12	0.01 (−0.15, 0.16)	0.94	−0.08 (−0.23, 0.08)	0.32	0.12 (−0.14, 0.27)	0.37	0.76
Restriction	3	0.15 (−0.02, 0.33)	0.09	0.08 (−0.09, 0.24)	0.37	−0.003 (−0.31, 0.40)	0.98	
	12	0.13 (−0.08, 0.35)	0.23	−0.07 (−0.29, 0.14)	0.5	0.12 (−0.22, 0.47)	0.48	0.41
Pressure	3	0.14 (−0.10, 0.38)	0.25	0.02 (−0.20, 0.24)	0.87	0.02 (−0.34, 0.38)	0.92	
	12	0.17 (−0.13, 0.47)	0.27	0.05 (−0.25, 0.34)	0.76	0.02 (−0.40, 0.44)	0.92	0.75
Monitoring	3	0.21 (−0.02, 0.43)	0.07	−0.06 (−0.27, 0.15)	0.59	−0.06 (−0.34, 0.23)	0.7	
	12	0.24 (−0.04, 0.51)	0.09	0.10 (−0.17, 0.37)	0.46	−0.18 (−0.54, 0.17)	0.31	0.23
	**Parenting**							
Warmth	3	0.03 (−0.08, 0.13)	0.64	0.09 (−0.01, 0.18)	0.08	−0.12 (−0.32, 0.07)	0.22	
	12	0.16 (0.00, 0.32)	0.05	0.19 (0.04, 0.35)	0.02 *	−0.10 (−0.33, 0.14)	0.42	0.69
Parent efficacy	3	−0.01 (−0.10, 0.08)	0.89	0.00 (−0.08, 0.08)	0.98	0.02 (−0.09, 0.12)	0.76	
	12	0.09 (−0.05, 0.23)	0.22	0.04 (−0.11, 0.18)	0.62	0.08 (−0.11, 0.27)	0.41	0.83
Self-efficacy	3	0.09 (−0.10, 0.27)	0.35	0.03 (−0.14, 0.20)	0.71	0.21 (−0.11, 0.52)	0.19	
	12	0.12 (−0.08, 0.32)	0.24	0.00 (−0.20, 0.19)	0.96	0.28 (−0.08, 0.63)	0.13	0.68
Inductive reasoning	3	−0.04 (−0.20, 0.12)	0.62	−0.07 (−0.22, 0.08)	0.37	−0.13 (−0.35, 0.09)	0.25	
	12	0.09 (−0.12, 0.30)	0.38	−0.04 (−0.24, 0.17)	0.73	−0.03 (−0.30, 0.24)	0.83	0.68
Hostility	3	−0.21 (−0.50, 0.08)	0.16	−0.03 (−0.30, 0.24)	0.14	0.11 (−0.43, 0.64)	0.70	
	12	−0.36 (−0.73, 0.02)	0.07	0.04 (−0.34, 0.41)	0.85	−0.11 (−0.74, 0.52)	0.73	0.31
Over protection	3	−0.03 (−0.29, 0.24)	0.86	−0.29 (−0.28, 0.22)	0.82	−0.09 (−0.50, 0.30)	0.64	
	12	0.20 (−0.05, 0.45)	0.11	0.29 (0.06, 0.53)	0.02 *	−0.20 (−0.50, 0.11)	0.20	0.84

^a^ Time differences between (baseline—3 months) and (baseline—12 months) at 95% of confidence interval; Child feeding domains analysed using the CFQ [4]; Parenting style dimensions from the Longitudinal Study of Australian Children (Australian Institute of Family Studies) [37,42].

**Table 4:** Regression analysis of child feeding practices and parenting style dimensions of all participants across all time-points in Feeding Healthy Food to Kids Randomised Control Trial (*n* = 146).

Child Feeding Practices								
Parenting Style Dimensions	Responsibility	*p* Value	Restriction	*p* Value	Pressure to eat	*p* Value	Monitoring	*p* Value
Warmth	0.13 (0.02, 0.24)	0.02 *	−0.02 (−0.13, 0.09)	0.69	−0.06 (−0.17, 0.00)	0.33	0.15 (0.05, 0.26)	0.01 *
Parental Efficacy	0.00 (−0.12, 0.12)	0.98	−0.03 (−0.15, 0.08)	0.58	0.08 (−0.04, 0.19)	*p* = 0.20	0.07 (−0.05, 0.18)	0.25
Inductive Reasoning	0.14 (0.03, 0.25)	0.02 *	−0.03 (−0.15, 0.08)	0.58	0.13 (0.01, 0.24)	*p* = 0.03 *	0.15 (0.03, 0.26)	0.01 *
Self-efficacy	−0.09 (−0.20, 0.03)	0.15	0.04 (−0.07, 0.16)	0.47	−0.10 (0.02, 0.02)	*p* = 0.10	−0.12 (−0.03, 0.00)	0.06
Hostility	−0.10 (−0.22, 0.01)	0.08	0.08 (−0.04, 0.19)	0.19	−0.06 (−0.18,0.05)	*p* = 0.29	−0.07 (−0.19, 0.04)	0.22
Overprotection	0.00 (−0.12, 0.11)	0.98	−0.02 (−0.14, 0.10)	0.73	0.08 (−0.04, 0.19)	*p* = 0.21	−0.02 (−0.13, 0.10)	0.76

* *p* < 0.05 reported as Pearsons Correlation (95% Confidence Interval).

**Table 5:** Correlation between child feeding and parenting style domain measures from baseline to 3 and 12 month in the FHFK RCT using correlation analysis (*n* = 146).

	RESP	REST	PTE	MON	W	PE	IR	H	SE	OP
Baseline to 3 months	0.572 ***	0.712 ***	0.634 ***	0.258	0.662 ***	0.314 *	0.576 ***	0.761 ***	0.716 ***	0.601 ***
Baseline to 12 months	0.568 ***	0.574 ***	0.545 **	0.124	0.452 **	0.225 *	0.483 **	0.725 ***	0.718 ***	0.226

* *p* < 0.05, ** *p* < 0.01, *** *p* < 0.0001 (adjusted for type 1 error using Bonferroni calculation); RESP: responsibility, REST: restriction, PTE: pressure to eat, MON: monitoring, W: warmth, PE: parental efficacy, IR: inductive reasoning, H: hostility, SE: self-efficacy, OP: overprotection; FHFK RCT: Feeding Healthy Food to Kids Random Control Trial.

**Table 6:** Correlation between child feeding scores over 12 months and parenting styles scores over 12 months and between child feeding score and parenting style score for all time points of the Feeding Healthy Food to Kids Randomised Control Trial (*n* = 146).

Domain	Child Feeding Score (3 Months)	Child Feeding Score (12 Months)	Parenting Style Score (3 Months)	Parenting Style Score (12 Months)	Parenting Style Score (*n* = 314)
Child feeding score baseline	0.56 (0.41, 0.67) **	0.47 (0.28, 0.2) **			
Parenting style score baseline			0.45 (0.28, 0.58) **	0.22 (0.00, 0.42)	
Child feeding score (total)					0.42 (0.33, 0.51) **

** *p* < 0.001 reported as Spearman’s Correlation (95% Confidence Interval) (adjusted for type 1 error using Bonferroni calculation). Random effects modelling of group-by-time within the FHFK RCT did not find an intervention effect for the child feeding score (*p* = 0.44) or parenting style score (*p* = 0. 70).
